# Extinction and Retrieval + Extinction of Conditioned Fear Differentially Activate Medial Prefrontal Cortex and Amygdala in Rats

**DOI:** 10.3389/fnbeh.2015.00369

**Published:** 2016-01-22

**Authors:** Hongjoo J. Lee, Rebecca P. Haberman, Rheall F. Roquet, Marie-H. Monfils

**Affiliations:** ^1^Department of Psychology, The University of Texas at AustinAustin, TX, USA; ^2^Department of Psychological and Brain Sciences, The Johns Hopkins UniversityBaltimore, MD, USA; ^3^Center for Learning and Memory, The University of Texas at AustinAustin, TX, USA

**Keywords:** fear conditioning, *Arc* catFISH, extinction, reconsolidation, retrieval + extinction

## Abstract

Pairing a previously neutral conditioned stimulus (CS; e.g., a tone) to an aversive unconditioned stimulus (US; e.g., a footshock) leads to associative learning such that the tone alone comes to elicit a conditioned response (e.g., freezing). We have previously shown that an extinction session that occurs within the reconsolidation window (termed retrieval + extinction) attenuates fear responding and prevents the return of fear in Pavlovian fear conditioning (Monfils et al., 2009). To date, the mechanisms that explain the different behavioral outcomes between standard extinction and retrieval + extinction remain poorly understood. Here we sought to examine the differential temporal engagement of specific neural systems by these two approaches using *Arc* catFISH (cellular compartment analysis of temporal activity using fluorescence *in situ* hybridization (FISH)). Our results demonstrate that extinction and retrieval + extinction lead to differential patterns of expression, suggesting that they engage different networks. These findings provide insight into the neural mechanisms that allow extinction during reconsolidation to prevent the return of fear in rodents.

## Introduction

Fear conditioning is a widely used paradigm in which the pairing of an initially neutral conditioned stimulus (CS) with an aversive unconditioned stimulus (US) leads to associative learning, such that when later presented with the CS alone, individuals will show a conditioned response (e.g., freezing). After conditioning, fear memories become strengthened over time through a process called consolidation (McGaugh, 2000). Once consolidated, fear memories are extremely persistent, and less susceptible to disruption. Two paradigms (blockade of reconsolidation and extinction) have traditionally been used in the laboratory setting to reduce acquired fear (Wolpe, 1969; Nader et al., 2000). In reconsolidation blockade, retrieval of a consolidated memory followed by pharmacological disruption (e.g., protein synthesis inhibition) leads to a sustained decrease in fear expression. In extinction, the repeated presentation of the CS in the absence of a US leads to a progressive decrease in fear expression (Pavlov, 1927; Rescorla and Heth, 1975; Robbins, 1990). The clinical efficacy of these techniques, however, has been limited. Reconsolidation blockade generally requires potentially toxic drugs, and extinction is not typically permanent (Rescorla and Heth, 1975; Bouton and Bolles, 1979a,b; Robbins, 1990). We devised an effective, drug-free paradigm for the persistent reduction of learned fear that capitalizes on the mechanistic differences between reconsolidation and extinction (Monfils et al., 2009). More specifically we applied extinction training during the retrieval-induced labile period to incorporate, during the reconsolidation window, the re-encoding of the CS as less threatening (retrieval + extinction). Using this approach, we were able to prevent the return of fear. Our retrieval + extinction paradigm has since been used successfully to persistently modify aversive and appetitive memories in rodents (Monfils et al., 2009; Rao-Ruiz et al., 2011; Xue et al., 2012; Olshavsky et al., 2013a,b; for a review, Auber et al., 2013; but see also: Chan et al., 2010). The effect has also been observed in humans (Schiller et al., 2010, 2013; Xue et al., 2012). It should be noted however that the phenomenon may be susceptible to boundary conditions which may not be fully understood at this point (for example, see Sevenster et al., 2012, 2014).

We previously showed that fear memory retrieval leads to increased levels of phosphorylated GluR1-containing AMPARs (pGluR1-containing AMPARs). When a second CS is presented 1 h after the initial retrieval, the receptors undergo dephosphorylation, possibly suggesting that destabilization of the memory trace might underlie the lack of fear reemergence in the retrieval extinction manipulation (Ret + Ext; Monfils et al., 2009). Clem and Huganir (2010) found that a central component of Ret + Ext-induced reduction in fear expression is the synaptic removal of CP-AMPARs in the lateral amygdala (LA), a metabotropic GluR1 receptors (mGluR1) dependent mechanism that leads to memory destabilization and subsequent reconsolidation, and an ensuing weakening of pre-existing synapses similarly to what occurs following long-term depression (LTD). Clem and Huganir (2010) thus showed that reconsolidation update and CP-AMPARs-mediated LTD share a requirement for mGluR1 activation. Recently, we observed a differential pattern of Zif268 and rpS6P expression in the amygdala and medial prefrontal cortex (mPFC) following extinction vs. retrieval + extinction. Those data suggested that new information from extinction training applied after the retrieval of a consolidated fear memory led to an updating in a reconsolidation process (Tedesco et al., 2014).

Still, to-date, the precise dynamic mechanisms underlying the different behavioral outcomes of standard extinction vs. extinction applied after an isolated retrieval are not completely understood. Here, we sought to examine the differential temporal engagement of specific neural systems by the initiation of Extinction vs. Retrieval + Extinction mechanisms, using *Arc* catFISH [cellular compartment analysis of temporal activity using fluorescence *in situ* hybridization (FISH)]. catFISH provides a brain-wide visualization of the populations of neuron that are selectively involved in two temporally-distinct events as identified by the presence of *Arc* mRNA either in a cell’s nucleus and/or cytoplasm (Guzowski et al., 1999; Vazdarjanova et al., 2002). We specifically quantified nuclear and cytoplasmic *Arc* expression in the amygdala (lateral and basal), and mPFC (prelimbic and infralimbic), as these regions have been implicated in fear consolidation and extinction (Phillips and LeDoux, 1992; Quirk et al., 1997; Knapska and Maren, 2009).

## Materials and Methods

### Animals

Male Sprague Dawley rats (250–300 g at arrival; Harlan Lab Animals Inc., IN, USA) were housed in pairs in clear plastic cages with food and water provided *ad libitum*. The rats were maintained on a 12 h light-dark cycle (lights on at 7 am) and the behavioral procedures were conducted during the light cycle. Procedures were conducted in compliance with the National Institutes of Health Guide for the Care and Use of Experimental Animals and were approved by the University of Texas at Austin Animal Care and Use Committee.

### Apparatus

All behavioral procedures took place in standard conditioning chambers made with stainless-steel walls and rod floors connected to a shock generator (Coulbourn Instruments, Allentown, PA, USA). Chambers were enclosed in acoustic isolation boxes (Coulbourn Instruments) and lit with a red light. Behavior was recorded with digital cameras mounted on the top of each unit. The chambers were wiped with soap and water between each session. Stimulus delivery was controlled using Freeze Frame software (Coulbourn Instruments). The CS was a tone (5 kHz, 80 dB) 20 s in duration and the US was a 0.7 mA foot-shock 500 ms in duration.

### Behavioral Procedure

The rats were first fear conditioned. After a 10 min habituation period in the chamber, rats received three presentations of the CS co-terminating with the US, with an average of 180 s intertrial intervals (ITI). The next day, the rats were divided to either (1) “1 then 4 CSs” or (2) “10 CSs” groups. The rats in the 1 then 4 CSs group (*n* = 6) received a single 20 s CS presentation in the absence of the US and were returned to their home cages in the colony for 15 min. Then, they were returned back to the chambers and received four more 20 s CS presentations without US (150 s ITI). The rats in the 10 CSs group (*n* = 6) received 10 20 s CS presentations in the absence of the US (150 s ITI). In addition to these two groups, a third group of rats (termed “1 then 4 tones”, *n* = 3) underwent mock fear conditioning the first day in which they were exposed to the three presentation of CS but not the accompanying US. The next day, they received an identical procedure as the 1 then 4 CSs group, in which they received one 20 s tone presentation followed by a 15 min period in the home cage, and then four additional presentations of 20 s tone. The behavioral procedures for all three groups lasted 30 min. These behavioral procedures were temporally arranged to detect expression of nuclear and cytoplasmic *Arc* mRNA, which have time-limited appearance in the activated neurons (Vazdarjanova et al., 2002). As seen in Figure 2, neurons activated during the first 5 min of the session should show peak cytoplasmic *Arc* expression at the time of perfusion (which occurred 30 min later) while neurons activated during the last 5 min of the session should show peak nuclear *Arc* expression at the time of perfusion which occurred immediately after the session was over. Neurons activated at both time points should have both cytoplasmic and nuclear *Arc* staining.

An experimenter blind to the overall hypothesis and design of the study scored freezing behavior manually from video recorded during each session. However, it was difficult for the experimenter to remain completely blind to the second day behavioral procedures in which the number of CS presentations differed between groups. Freezing was defined as the absence of any movements, excluding those required for respiration. The total number of seconds spent freezing throughout the CS presentation was expressed as a percentage of CS duration.

### Histology Procedure

Immediately after the end of the 30 min behavioral procedure on the second day, rats received an overdose of pentobarbital (86 mg/kg) and phenytoin (11 mg/kg) mix (Euthasol^®^ by Virbac Animal Health) and then were perfused transcardially with 0.9% saline followed by 4% Paraformaldehyde (PFA) in 0.1 M phosphate buffer (PB). The brains were extracted, placed into a 20% sucrose PFA/PB solution overnight, rapidly frozen using powdered dry ice the next day and stored at −80°C. The brains were sliced as 25 μm thick coronal sections using a sliding microtome and the sections were immediately mounted on slides. Then, they were vacuum dried overnight at room temperature and stored in an air-tight container with desiccant at −80°C.

### Fluorescence *in situ* Hybridization

Every fifth section containing the medial PFC and the amygdala were processed with FISH for *Arc* mRNA detection using a modified protocol of Petrovich et al. (2005). Slides were treated with proteinase K and then with acetic anhydride. Then, they were gradually dehydrated through ascending concentrations of ethanol solutions. The sections were then covered with hybridization solution containing cRNA probe and incubated for 20 h at 60°C. The cRNA riboprobe was generated by using T7 RNA polymerase (Ambion; Grand Island, NY, USA) and by incorporating digoxigenin-UTP (DIG RNA labeling mix; Roche Applied Science, Indianapolis, IN, USA). The riboprobe was then purified using mini Quick Spin Columns (Roche). The plasmid used for generating *Arc* antisense contained the full length cDNA (~3.0 kbp) of *Arc* transcript.

After hybridization, slides were first washed in 4X SSC at 60°C before being treated with RNase and then washed in descending concentrations of SSC at 60°C. Then, the slides underwent immunocytochemical process using the PerkinElmer Tyramide Signal Amplification system (NEL704A; PerkinElmer, Waltham, MA, USA). Briefly, the tissue was incubated with anti-digoxigenin conjugate for 2 h and with cyanine 3 substrate for 30 min. Then, the tissue was covered slipped using a mounting medium that contained the nuclear stain 4′, 6-diamidino-2-phenylinodole, DAPI (Vectashield; Vector Lab, Burlingame, CA, USA).

### Image Acquisition and Analysis

Images were acquired using a fluorescence laser scanning confocal microscope, Zeiss LSM 710 (Zeiss: Thornwood, NY, USA). First, the correct regions of interest [i.e., prelimbic and infralimbic cortices, and lateral and basal amygdala (BA)] were identified based on nuclear DAPI staining with 10× objective. Then, using 40× oil objective, confocal *z*-stacks composed of 0.9 μm thick optical sections were collected through the regions of interest. A typical confocal stack had ~12 optical sections that contained ~112 cells identified by nuclear DAPI staining. For each of the prelimbic and infralimbic regions, an average of six stacks were collected from the sections that were between 3.20 to 2.80 anterior to Bregma according to Brain Maps v3 (Swanson, 2004). For Amygdala, an average of eight stacks in the lateral nucleus and four stacks in the basal nucleus were collected between 1.78 to 2.45 posterior to Bregma (Swanson, 2004).

Using Imaris software (Bitplane; Concord, MA, USA), an experimenter blind to the behavioral conditions analyzed the acquired images. Only the cells that showed the entire nuclei DAPI staining throughout the *z*-sections were considered. First, the cells that contained diffused perinuclear *Arc* staining were counted and classified as “cytoplasm”. Second, the cells that contained clear two *Arc* intranuclear foci were counted and classified as “nucleus”. Then, the cells that contained both the perinuclear and intranuclear foci staining of *Arc* were classified as “double”. These *Arc*+ cells were calculated as percentage of the overall DAPI stained cells for each stack and then averaged across the sampled stacks.

### Statistical Analysis

Statistical analyses were carried out using SPSS Statistics software. One-way ANOVAs with retrieval group as between subject factors were conducted. Where appropriate, *post hoc* tests were performed with Tukey’s honestly significant difference mean comparison.

## Results

### Retrieval and Extinction of Conditioned Fear

Our previous work showed that extinction and retrieval + extinction procedures led to different behavioral outcomes. Here, we sought to determine how the two differ at the timepoint where we hypothesize they mechanistically diverge. Two principal groups were run for this experiment. The first group (1 then 4 CSs) was representative of the initiation of the retrieval + extinction memory updating and associated mechanisms. The second group (10 CSs) was representative of the initiation of extinction and associated mechanisms.

**Figure 1:**
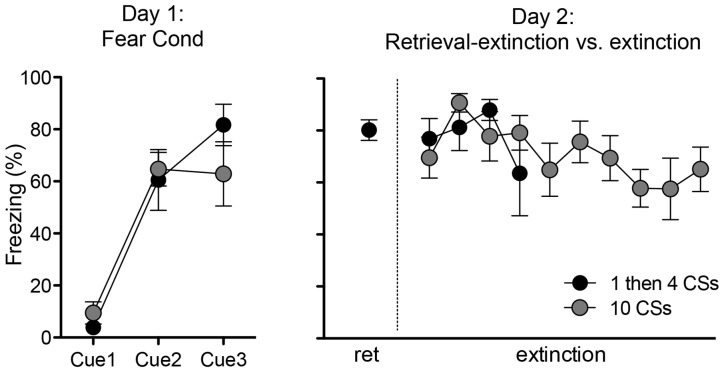
**Freezing during fear conditioning with tone-shock pairings on Day 1 and tone presentations on Day 2.** Rats in both groups showed fear acquisition and displayed comparable freezing levels on Day 1. On Day 2, rats in the 1 then 4 CSs group (*n* = 6) received a single tone presentation and then four additional tone presentations 15 min later to initiate the retrieval-extinction session, while rats in the 10 CSs group (*n* = 6) received 10 tone presentations to initiate the standard extinction session.

The rats in these two groups (“1 then 4 CSs” and “10 CSs”) received an identical fear conditioning procedure (i.e., three pairings of tone CS—shock US) on the first day. As expected, there was no difference in freezing during the fear conditioning session between the two groups (Figure 1 left panel). One-way ANOVA with repeated measures over three trials show a significant within-subjects effect, *F*_(2,20)_ = 39.4, *p* < 0.001, indicating that rats froze significantly more toward the end compared to the beginning of the session. And there was no main effect of groups, *F*_(1,10)_ = 0.17, *p* > 0.5, supporting that both groups of rats displayed comparable freezing.

**Figure 2:**
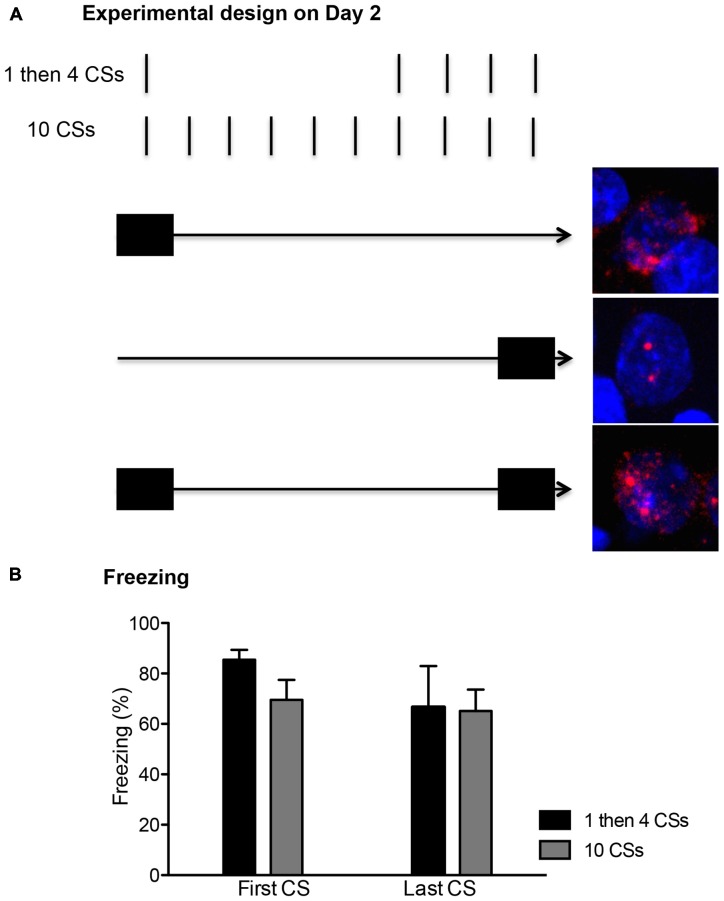
**(A)** The conditioned stimulus (CS) presentations were arranged temporally to correlate with the peaks for cytoplasm and nuclear expression of *Arc* mRNA. The first CS presentation in both groups occurred within the first 5 min (depicted with a filled rectangle). This allowed for about 25 min wait period since the initial *Arc* induction, showing peak cytoplasmic expression (shown as the red perinuclear staining around the blue DAPI+ cell in the top picture). The last CS presentation in both groups occurred within the last 5 min before the rats were killed, thus matching the peak nuclear *Arc* expression (shown as two red foci inside of DAPI+ cell in the middle picture). A cell with *Arc* induction at both time points should show both nuclear and perinuclear staining as seen in the bottom picture. **(B)** Freezing levels were comparable between the first and last CS presentations and also between the two groups (*n* = 6 for each group).

On the second day, the rats returned to the conditioning chambers and received either: (1) a single presentation of CS followed by 15 min in the homecage and then four additional CS presentations in the chamber (1 then 4 CSs group); or (2) ten CS presentations (10 CSs group). All rats showed significant conditioned freezing to the CS and the levels were similar between the groups (Figure 1 right panel). One-way ANOVA with repeated measures over the first 4 trials of extinction show no main effect of groups, *F*_(1,10)_ = 0.12, *p* > 0.5. Furthermore, we compared conditioned freezing during the very first CS exposure and the last CS exposure of these two groups given that the behavioral procedure was designed to detect two different time points of neuronal activation (i.e., the first and last 5 min) using the catFISH method (Figure 2). Thus, for the 1 then 4 CSs group, conditioned freezing is shown from the single CS presentation prior to 20 min homecage time and from the fourth CS presentation given after the homecage time. For the 10 CSs group, conditioned freezing is shown from the first and tenth CS presentations. The freezing levels were not different between the groups neither during the first CS, *t*_(10)_ = 1.8, *p* > 0.1, nor during the last CS, *t*_(10)_ = 0.1, *p* > 0.5. We expected to see activation of *Arc* during the initial 5 min with the first CS primarily in cytoplasm and *Arc* activation during the last 5 min with the last CS primarily in the nucleus.

### Detection of *Arc* mRNA Activated by Fear CS

Using nuclear DAPI staining as an anatomical guide, four regions of interest (i.e., prelimbic and infralimbic regions of the mPFC, and lateral and basal nuclei of the amygdala) were analyzed via confocal *z*-stacks. Then, DAPI-stained cells that expressed nuclear and/or cytoplasmic *Arc* were calculated.

**Figure 3:**
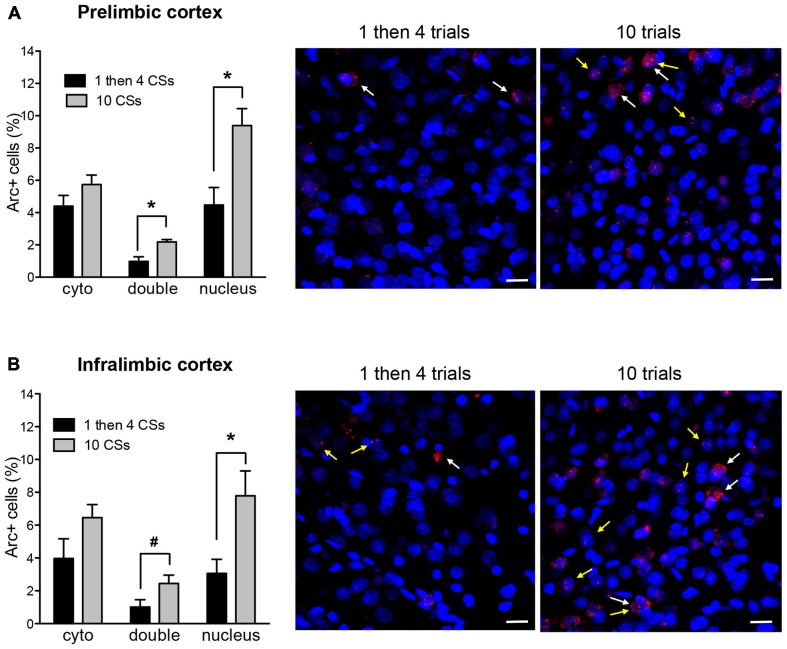
**The bar graphs show percentage of DAPI+ cells expressing *Arc* mRNA in the cytoplasm (cyto), nucleus, or both (double) in the prelimbic cortex (PL) (A) and infralimbic cortex (IL) (B).** The photomicrographs are maximum intensity projections of representative *z*-stacks from the sampled regions in the medial prefrontal cortex (mPFC). DAPI+ cells are shown in blue and *Arc* mRNA are shown in red. The yellow arrows point to *Arc* foci in the nuclei and the white arrows point to the *Arc* in the perinuclear areas (cytoplasm). Scale bar = 20 μm. *p < 0.05, ^#^*p* = 0.058.

Figure 3 shows *Arc*+ cells in the prelimbic **(A)** and infralimbic **(B)** areas between the two groups with representative photomicrographs. For both regions, there was no difference in cytoplasmic *Arc* expression between the two groups (*p*’s > 0.1 for both). This suggests that there were comparable neuronal activation by the initial CS presentation at the beginning of the session. However, there was a significant difference in nuclear *Arc* expression in which the rats in the 10 CSs group showed significantly more nuclear staining both in the prelimbic cortex (PL; *t*_(10)_ = 3.25, *p* < 0.01) and in the infralimbic cortex (IL; *t*_(10)_ = 2.72, *p* < 0.05). This suggests that more neurons were activated by the CS presentation during the last 5 min among the rats in the 10 CSs group. Furthermore, there were also more double labeled cells in the 10 CSs group both in the PL (*t*_(10)_ = 3.67, *p* < 0.01) and in the IL (*t*_(10)_ = 2.14, *p* = 0.058). This suggests that the neurons initially engaged by the CS presentation at the beginning of the 30 min session were recruited again by the CS presentation at the end of the session.

**Figure 4:**
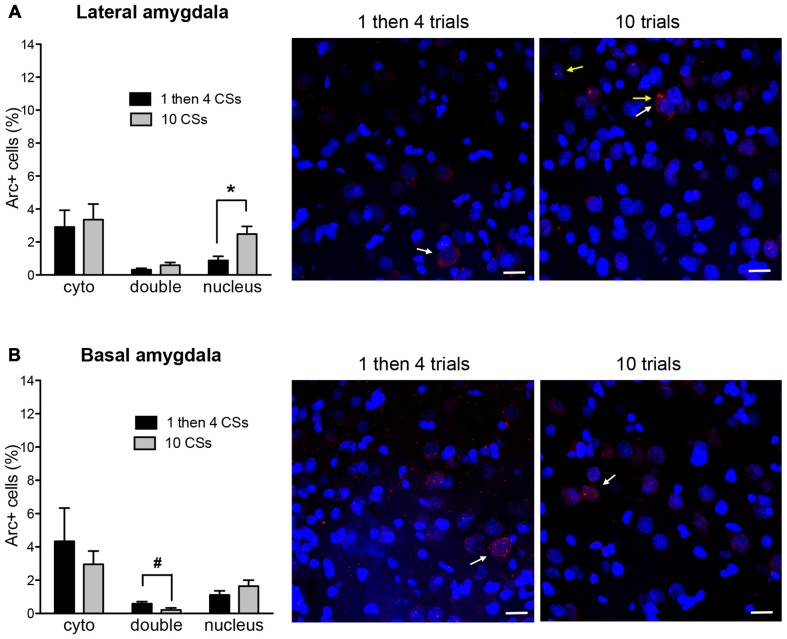
**The bar graphs show percentage of DAPI+ cells expressing *Arc* mRNA in the cytoplasm (cyto), nucleus, or both (double) in lateral nucleus (A) and basal nucleus (B) of the amygdala.** The photomicrographs are maximum intensity projections of representative *z*-stacks from the sampled regions in the amygdala. DAPI+ cells are shown in blue and *Arc* mRNA are shown in red. The yellow arrows point to *Arc* foci in the nuclei and the white arrows point to the *Arc* in the perinuclear areas (cytoplasm). Scale bar = 20 μm. *p < 0.05, ^#^*p* = 0.054.

Figure 4 shows *Arc*+ cells in the lateral **(A)** and basal **(B)** nuclei of the amygdala between the two groups with representative photomicrographs. Within the LA, there was no difference in cytoplasmic *Arc* expression between the two groups (*p* > 0.5). This suggests that there was comparable neuronal activation by the initial CS presentation. However, there was a significant difference in nuclear *Arc* expression in which the rats in the 1 then 4 CSs group showed significantly reduced nuclear staining compared to the 10 CSs group (*t*_(10)_ = 3.05, *p* < 0.05). This suggests that fewer neurons were activated by the CS presentation during the last 5 min among the rats in the 1 then 4 CSs group. Unlike in the LA, there were no obvious group differences in the BA with the exception of the marginally significant difference seen with double labeled cells, *t*_(10)_ = 2.19, *p* = 0.054.

**Figure 5:**
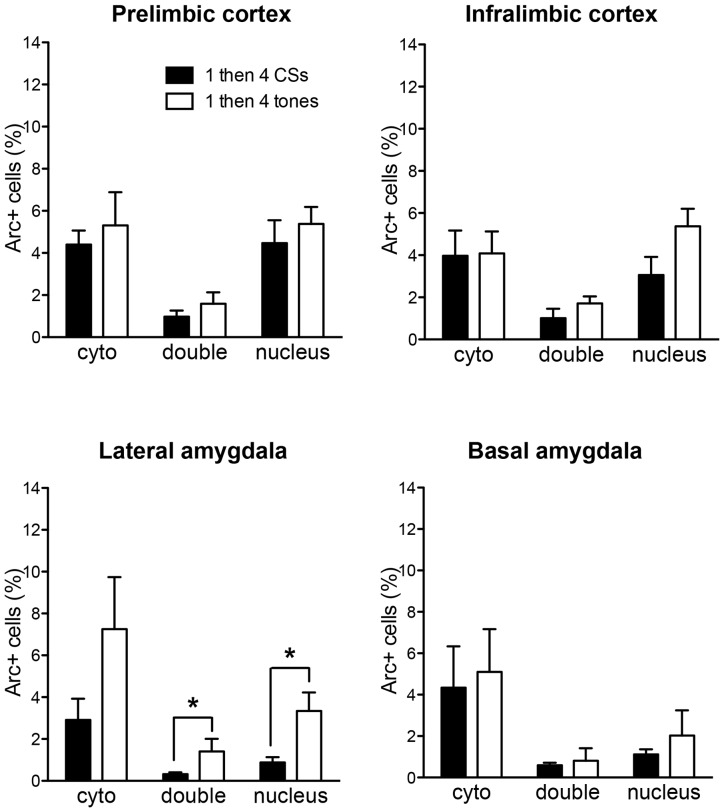
**The bar graphs show percentage of DAPI+ cells expressing *Arc* mRNA in the cytoplasm (cyto), nucleus, or both (double) of the 1 then 4 CSs group (*n* = 6) shown in Figures 2, 3 and the non-fear conditioned control group (1 then 4 tones, *n* = 3) that received identical CS presentations.** *p < 0.05.

In order to rule out the possibility that these differences might be purely based on the differences in the number of CS presentations, *Arc* expression of the 1 then 4 CSs group was compared to a third group (i.e., 1 then 4 tones group) that received mock fear conditioning (i.e., 3 CS presentations without US) and an identical procedure as the 1 then 4 CSs group on the second day. Figure 5 shows the comparisons of *Arc* expression in all four regions of interest. The *Arc* expression of the control (1 then 4 tones) group was similar to 1 then 4 CSs group in both subregions of the mPFC as well as the BA. In terms of the LA, there was no significant difference in the cytoplasm *Arc* (*t*_(7)_ = 1.97, *p* = 0.089); however, there was a significant difference in the nucleus *Arc* staining. There were fewer activated cells in the 1 then 4 CSs group than the control (1 then 4 tones) group (*t*_(7)_ = 2.55, *p* < 0.05). Furthermore, there were significantly fewer double-labeled cells in the 1 then 4 CSs (*t*_(7)_ = 2.64, *p* < 0.05) suggesting that, relative to the 1 then 4 tones, a fewer portion of the cells that were engaged by the initial CS presentation were recruited again by the CS presentations at the end of the session.

## Discussion

Memories acquired through fear conditioning are extremely persistent. Extinction and reconsolidation blockade are routinely used in laboratory settings to attenuate fear memories, though their clinical efficacy remains limited. Reconsolidation-based interventions are generally effective in permanently modifying memories, but they often require the use of toxic drugs that are not safe for use in humans (propranolol being a notable exception, Kindt et al., 2009). Extinction-based approaches (e.g., exposure therapy) do not work in all individuals, and for those in which they are effective, fear re-emergence often occurs. We, and others, previously showed that a combination of these two approaches, that is, extinction applied after retrieval of consolidated memories, prevented fear reemergence and drug-seeking relapse in a context-independent way in rats and humans (Monfils et al., 2009; Schiller et al., 2010, 2013; Rao-Ruiz et al., 2011; Xue et al., 2012; Olshavsky et al., 2013a,b; for a review, Auber et al., 2013; see also Sevenster et al., 2012, 2014). To date, cellular and molecular mechanisms underlying extinction applied during reconsolidation remain poorly understood.

The present study examined the differential temporal engagement of specific neural systems by the initiation of Extinction vs. Retrieval + Extinction mechanisms, using *Arc* catFISH. Two main experimental groups were conducted: an extinction mechanism group (10 CSs) and a retrieval + extinction mechanism group (1 then 4 CSs). The 10 CSs group was chosen to allow us to examine the circuitry engaged as extinction mechanisms begin to be progressively recruited. The 1 then 4 CSs group was chosen to isolate the circuitry engaged as mechanisms associated with retrieval + extinction (extinction applied after an isolated retrieval) are recruited. We examined 4 brain regions: IL, PL, LA, and BA. These regions were selected, because previous studies found them to be engaged during fear extinction (Quirk et al., 1997; Knapska and Maren, 2009). Knapska and Maren (2009) previously showed that reduced fear expression in response to a CS in the extinction context is associated with increased activity in the IL, and the return of fear to a CS presented in a different context is associated with activity in PL and LA.

Importantly, we found that the 10 CSs and the 1 then 4 CSs groups showed significantly different overall patterns of *Arc* expression as the mechanisms of extinction and retrieval + extinction became progressively initiated. The initial neural engagement in the prefrontal cortex was comparable in our two experimental groups—there was no difference in *Arc* expression in the cytoplasm in the PL and IL. The involvement of these brain structures intensified in the 10 CSs group, suggesting the continued and increasing engagement of the IL as extinction processes were recruited (in line with previously reported findings from the literature, Quirk et al., 1997; Milad and Quirk, 2002; Knapska and Maren, 2009; Do-Monte et al., 2015). The continued engagement of the PL in our 10 CSs group is likely reflective of the maintained behavioral fear response at this stage of the extinction protocol. PL has previously been found to be required for fear expression, and not to be required for the maintenance of extinction (Sierra-Mercado et al., 2011). Our results further suggest that a portion of the cells activated during the later phase of our 10 CSs group were newly recruited as extinction progressed, as evidenced by the fact that only a fraction of the cells in these regions expressed double labeling in both nucleus and cytoplasm. A different pattern emerged in the 1 then 4 group. Fewer cells were *de novo* recruited in the latter phase of this experimental group, as evidenced by the fact that there was significantly less nuclear staining than in the 10 CSs group. Furthermore, only very few cells showed double labeling.

The two groups also differed in their expression in the amygdala. Similarly to what was observed in the prefrontal cortex, the two groups were comparable in cytoplasmic staining for both the LA and the BA at the beginning of their respective experimental window. The 1 then 4 CSs group showed fewer cells with nuclear staining relative to the 10 CSs group, in the face of comparable double expression, suggesting that while the 10 CSs group recruited more cells in the LA during the later phase of the extinction paradigm, the 1 then 4 groups did not. There were no differences in BA.

Together, our results in the prefrontal cortex and amygdala indicated that the initiation of reconsolidation updating (retrieval + extinction) differed from that of the initiation of standard extinction. We next compared the results from our 1 then 4 CSs group to that of a group that received the same pattern of Cue (tone) presentations, but which had not been fear conditioned the previous day. Our results show no difference between our two groups in the IL, PL, and BA, suggesting little engagement of these structures by the retrieval extinction manipulation beyond baseline levels. Interestingly, there were notable differences in the LA, whereby there were fewer cells expressing double and nuclear staining in the 1 then 4 CSs (conditioned group) than its control (unconditioned) counterpart. These results help solidify the notion that fewer cells in the LA were activated as retrieval-extinction mechanisms engaged, compared to what occurs in the case of standard extinction, and that difference observed between the 10 CSs and the 1 then 4 CSs groups was not simply due to a difference in the number of tone presentation.

*Arc* is thought to play a critical role in synaptic plasticity (Guzowski et al., 2000; Plath et al., 2006; Messaoudi et al., 2007), and in the present study, identifies the neurons that are active in response to different groupings of CS presentations, with the advantage of capturing the neural ensembles that are involved at two different time points. The LA results appear to be in-line with our previously published findings (Monfils et al., 2009). Effectively, we previously found that either a single CS, or 2 CSs presented with an interval of 3 min (akin to intervals typically used in a standard extinction paradigm) led to an increase in GluR1 expression in the LA. When the CS was applied 1 h after an isolated CS led to a dephosphorylation of GluR1 receptors in the LA (Monfils et al., 2009). Together with the present results, as well as the findings of Clem and Huganir (2010), we propose that the retrieval + extinction may occur through an active reversal of plasticity in the LA.

More recently, we examined the effects of extinction vs. retrieval + extinction on the expression of two different proteins (zinc-finger protein 268 [zif268], and phosphorylated ribosomal protein S6) in the IL, PL, LA and CA1 region of the hippocampus. The experiments from that study revealed that extinction applied after retrieval selectively increased zif268 and phosphorylated ribosomal protein S6 in the prefrontal cortex and amygdala, in a pattern of activity that was distinct from standard extinction (Tedesco et al., 2014).

Taken together, these studies suggest that at the beginning, as well as the end of training, extinction and retrieval + extinction engaged divergent brain mechanisms. In the present study, we used a more dynamic approach (*Arc* catFISH), which allowed us to identify the networks engaged as a result of extinction vs. retrieval + extinction at the time-point where we believe the two protocols to mechanistically diverge. Effectively, we hypothesized that the two protocols would lead to comparable circuit activation at the beginning of training, which generally corresponds to memory retrieval. For the first time, we were also able to determine which cells, of those that were active near the end of our training paradigms, were also active at the beginning (fear retrieval timepoint), and which were *de novo* recruited. The latter was crucial in allowing us to determine whether increased activity would be best explained as sustained increased engagement from fear memory retrieval, recruitment of new cells, or a combination of both.

Our data reveal the differential engagement of amygdala, and mPFC subregions during extinction vs. retrieval + extinction, thereby highlighting their specific dynamic contributions at the moment where their mechanistic contributions are thought to diverge. In essence, our results strengthen the notion that extinction applied during the reconsolidation window engages mechanisms distinct from standard extinction, and explains why they lead to drastically different behavioral outcomes.

## Author Contributions

HJL and M-HM designed, conducted and wrote the work. RPH provided *Arc* plasmid and helped with designing the procedure for fluorescence *in situ* hybridization. RFR helped with behavioral experiment and data analyses.

## Conflict of Interest Statement

The authors declare that the research was conducted in the absence of any commercial or financial relationships that could be construed as a potential conflict of interest.
